# *Mori Cortex Radicis* Attenuates High Fat Diet-Induced Cognitive Impairment via an IRS/Akt Signaling Pathway

**DOI:** 10.3390/nu12061851

**Published:** 2020-06-21

**Authors:** SoHyeon You, Miran Jang, Gun-Hee Kim

**Affiliations:** 1 Department of Health Functional Materials, Duksung Women’s University, Seoul 01369, Korea; ush0206@hanmail.net; 2 Department of Food Science, Purdue University, West Lafayette, IN 47906, USA; jang126@purdue.edu; 3 Department of Food and Nutrition, Duksung Women’s University, Seoul 01369, Korea

**Keywords:** *Mori Cortex radicis*, high-fat diet, obesity, cognitive impairment

## Abstract

Present study was conducted to investigate ameliorating effects of *Mori Cortex radicis* on cognitive impair and neuronal defects in HFD-induced (High Fat Diet-Induced) obese mice. To induce obesity, C57BL/6 mice were fed an HFD for 8 weeks, and then mice were fed the HFD plus *Mori Cortex radicis* extract (MCR) (100 or 200 mg/kg/day) for 6 weeks. Prior to sacrifice, body weights were measured, and Y-maze test and oral glucose tolerance test were performed. Serum lipid metabolic biomarkers (TG, LDL, and HDL/total cholesterol ratio) and antioxidant enzymes (glutathione, superoxide dismutase, and catalase), malondialdehyde (MDA), and acetylcholinesterase (AChE) levels were measured in brain tissues. The expressions of proteins related to insulin signaling (p-IRS, PI3K, p-Akt, and GLUT4) and neuronal protection (p-Tau, Bcl-2, and Bax) were examined. MCR suppressed weight gain, improved serum lipid metabolic biomarker and glucose tolerance, inhibited AChE levels and MDA production, and restored antioxidant enzyme levels in brain tissue. In addition, MCR induced neuronal protective effects by inhibiting p-Tau expression and increasing Bcl-2/Bax ratio, which was attributed to insulin-induced increases in the expressions p-IRS, PI3K, p-Akt, and GLUT4. These indicate MCR may reduce HFD-induced insulin dysfunction and neuronal damage and suggest MCR be considered a functional material for the prevention of T2DM-associated neuronal disease.

## 1. Introduction

Obesity is generally believed to be caused by excessive eating and environmental and genetic factors and is considered a low-grade inflammatory disease [1]. However, obesity is a component of metabolic disorders such as dyslipidemia, insulin resistance, hyperlipidemia, hypertension, and type 2 diabetes (T2DM) [2]. In particular, type 2 diabetes induces insulin resistance, oxidative stress, inflammation, and aging [3]. Insulin resistance (IR) is a symptom of dysfunctional glucose metabolism. It is the pathological characteristic of type 2 diabetes and occurs during the first stage of the disease [4]. In addition, IR precedes central nervous system dysfunction and neuronal diseases such as Alzheimer’s disease [5]. Recently, T2DM and insulin resistance were reported to be closely related to neurodegenerative diseases and cognitive disorders [6,7]. Diabetic patients have been reported to be at 50–75% higher risk of Alzheimer’s disease, and 80% of Alzheimer’s disease patients have been reported to have abnormal fasting glucose levels [8]. Therefore, neurodegenerative diseases have been described as the third type of diabetes or a brain insulin resistant state [5]. Insulin is critical for normal brain function and peripheral glucose metabolism. Insulin stimulates neurite growth in brain neuronal cells, releases and absorbs neurotransmitters like catecholamine, and continuously exerts neuronal excitement by regulating ion channels and synapse plasticity. It has also been suggested insulin dysfunction caused by obesity, diabetes, or cardiovascular disease might adversely influence brain neuronal functions [5]. Insulin resistance is an insulin dysfunction, in which insulin receptors do not function properly, which inactivates downstream components of the insulin pathway such as insulin receptor substrate (IRS). Furthermore, disruption of the insulin signaling inhibits the PI3K/Akt pathway, and over-phosphorylation of tau results in the accumulation of neurofibrillary tangles (NFTs) that induce neuron and brain neuronal cell apoptosis, which lead to neurodegenerative diseases like Alzheimer’s disease [5].

It has been established that a high-fat diet (HFD) increases insulin resistance and elevates oxidative stress in the brain [3]. A previous study demonstrated long-term consumption of an HFD disrupts insulin signaling in the hippocampus and blood glucose homeostasis and oxidative systems by inducing the overproduction of reactive oxygen species (ROS) [6]. It has also been reported diabetic states cause ROS over-production, damage brain neuronal cells, and result in cognitive disorders [9]. Furthermore, insulin disorders might also induce oxidative stress and brain neuronal cell apoptosis by inducing mitochondria malfunction [7].

*Mori Cortex radicis* is the root bark of *Moraceae* species like *Morus alba* L. and has been shown to have antipyretic, anticonvulsant, antiallergic, and anti-inflammatory effects. Traditionally, *Mori Cortex radicis* has been used as pharmacologic ingredient to treat diuresis and diabetes [10,11]. Several studies have reported *Mori Cortex radicis* contains antioxidant compounds such as stilbene, flavonoids, alkaloids, and Diel-Alder type adducts with anti-obesity, anti-diabetes, and anti-inflammatory effects [12,13,14]. We previously reported *Mori Cortex radicis* has radical scavenging and antioxidative effects and inhibits neuronal cell apoptosis in neuronal cells exposed to high glucose levels [10,11]. In the present study, we investigated whether an extract of *Mori Cortex radicis* affects p-IRS/PI3K/Akt insulin signaling and has neuro-protective effects in an HFD-induced mouse model of obesity.

## 2. Materials and Methods

### 2.1. Materials

*Mori Cortex radicis* (MCR) was cultivated in Andong (Kyeongbuk) and dried MCR was purchased in Kyeongdong Market (Seoul, Korea) in April 2017. Metaphosphoric acid, thiobarbituric acid, phosphoric acid, 5,5’-dithiobis-(2-nitrobenzoic acid), and sodium phosphate were purchased from Sigma-Aldrich Chemical Co. (St. Louis, MO, USA). Primary antibodies for P-IRS, IRS, PI3K, p-Akt, Akt, Bax, Tau, GLUT4, AChE, and β-actin were purchased from Cell Signaling Technology (Danvers, MA, USA), and p-Tau and Bcl-2 were acquired from Santa Cruz Biotechnology Inc. (Santacruz, CA, USA). Horseradish peroxidase (HRP)-conjugated anti-rabbit and anti-mouse (secondary antibody) were purchased from Bio-Rad Co. (Bio-Rad, Hercules, CA, USA). Enhanced chemiluminescence (ECL) solution was obtained from Amersham Life Science Corp. (Arlington Heights, IL, USA), and other chemicals were purchased from Sigma-Aldrich Chemical Co. (St. Louis, MO, USA).

### 2.2. Sample Preparation

Dried *Mori Cortex radicis* (30 g) was extracted twice with 300 mL of 70% ethanol for 6 h at 50 °C and filtered through filter paper (Toyo Kaisha Ltd., Tokyo, Japan). The filtrate so obtained was concentrated using a vacuum evaporator (EYELA N-1000, Riakikiai Co., Ltd., Tokyo, Japan) and freeze-dried (IlShin Co., Ltd., Yangju, Korea) for 48 h. Powdered *Mori Cortex radicis* extract (MCR) was stored at −20 °C until required and dissolved in sterile water prior to oral administration.

### 2.3. Animals and Study Design

The animal study was carried out in accordance with the guidelines issued by the Institutional Animal Care and Use Committee (IACUC) at Duksung Women’s University (approval no. 2019-010). Four-week-old male C57BL/6 mice were purchased from Orient Bio (Sungnam, Korea) and housed under controlled conditions (22 ± 2 °C; 50 ± 5% humidity) under a 12 h light/dark cycle. Mice were acclimated for one week and placed on an HFD (high-fat diet; 60% kcal fat) (*n =* 24) for 14 weeks. During the first 8 weeks on the HFD, obesity status was observed, and then, animals were randomly divided into three treatment groups, as follows: animals in the HFD group (*n =* 8) were administered HFD plus sterilized water; animals in the MCR100 group (*n =* 8) were administered the HFD plus 100 mg/kg of MCR daily, and animals in the MCR200 group (*n* = 8) were administered the HFD plus 200 mg/kg MCR daily for 6 weeks. Animals in the normal diet (ND) group (*n* = 8) were administered ND with sterilized water for 14 weeks. Body weights were measured weekly during the 14-week experimental.

### 2.4. Y-Maze Behavioral Test

The Y-maze apparatus was made of black-painted plastic material, and the length, height, and width of each arm were 33, 15, and 10 cm, respectively. For testing, a mouse was placed in the center of the apparatus and allowed to move freely for 8 min. Actual alternation was defined as the number of consecutive entries into the three arms (A, B, or C) [15] on overlapping triplet sets. Alternation behavior (%) was calculated as follows:

(1) $$\mathrm{Alternation}\;\mathrm{behavior}\;(\%)=\frac{\mathrm{actual}\;\mathrm{alternation}}{\mathrm{Total}\;\mathrm{number}\;\mathrm{of}\;\mathrm{arm}\;\mathrm{entries}-2}$$

### 2.5. Oral Glucose Tolerance Testing (OGTT)

After 14 weeks on their respective diets, oral glucose tolerance testing was performed. Mice were fasted for more than 12 h, and glucose (2 g/kg) was administrated by oral gavage [16]. Blood was drawn from mouse tails 0, 15, 30, 60, and 120 min later and tested using a commercial glucometer (Accucheck, Roche Diagnostic, Basel, Switzerland).

### 2.6. Serum and Brain Tissue Collection

Before sacrifice, mice were fasted for 12 h, anesthetized under CO2, blood samples were collected by cardiac puncture, left undisturbed at room temperature for 30 min, and centrifuged (Micro12 Hanil, Incheon, Korea) at 3000 rpm for 10 min at 4 °C. Supernatants were subjected to biochemical assay using an automatic analyzer (Hitachi 7180, Tokyo, Japan). Whole brains were quickly removed after blood sampling, and brain tissues were washed with PBS and stored at −70 °C until required for protein extraction.

### 2.7. Measurement of Malondialdehyde (MDA) Contents

Lipid peroxidation was quantified by measuring malondialdehyde (MDA) levels using the TBARS method. Briefly, whole brain tissues were homogenized with 20 mM tris-HCl buffer (pH 7.4) and centrifuged at 12,000 × g for 15 min at 4 °C. Supernatants were precipitated using phosphoric acid and incubated with thiobarbituric acid (TBA) at 95 °C for 1 h. Organic complexes were extracted; *n*-butanol and absorbances were measured at 532 nm using a microplate reader. Lipid peroxidation was quantified using an MDA (Sigma Co., St. Louis, MO, USA) calibration curve.

### 2.8. Measurement of Antioxidant Enzymes Activity

Superoxide dismutase (SOD) and glutathione (GSH) contents and catalase (CAT) activity in brain tissues were used to antioxidant enzyme activities. Extracted brain tissues were homogenized in PBS and spun down at 1200 g for 10 min at 4 °C. Assays were performed using the supernatants obtained. SOD contents were measured using a commercial SOD kit (Sigma-Aldrich Chemical Co., St. Louis, MO, USA), and results were expressed as unit per mg of protein. GSH contents were measured using a commercial GSH kit (Enzo Life Science Inc., Lausen, Switzerland) and expressed as nM per mg of protein. CAT activities were measured using a commercial CAT kit (Cayman Chemical, Ann Arbor, MI, USA) and expressed as nM per min per mL.

### 2.9. Acetylcholinesterase (AChE) Activity

To assess AChE activities and protein expressions, whole brains were homogenized with 10 mM Tris-HCl (pH 7.2) and centrifuged at 14,000 g for 30 min at 4 °C. Supernatants were subjected to assay. AChE activity was determined by determining the rate of hydrolysis of acetylcholine iodide in the presence of 100 mM phosphate buffer (pH 7.5) and 2 mM DTNB. Reactions were monitored at 412 nm every 30 secs for 2 min using a microplate reader. AChE activities were expressed as percentages of those of ND group.

### 2.10. Western Blot Analysis

Whole brain tissues were homogenized in protein extraction solution (Intron, Seoul, Korea) containing 1% protease inhibitor cocktail (Thermo Fisher Scientific, Rockford, IL, USA) using a tissue blender and centrifuged at 12,000 rpm for 20 min at 4 °C. To equalize total protein amounts, protein concentrations in supernatants were measured using the Bradford protein assay (BioRad Laboratories, Hercules, CA, USA). Proteins (30 μg) separated using SDS gels and then transferred to polyvinylidene difluoride (PVDF) membranes (Millipore, Billerica, MA, USA). After blocking with 5% skim milk in tris buffered saline containing Tween-20 (TBST), membranes were incubated overnight with the following primary antibodies at 4 °C; actin, p-IRS-1 (Tyr 612), IRS-1, PI3K, p-Akt (Ser 473), Akt, AChE, GLUT4, Bax, Bcl-2, p-Tau (Ser 404), and Tau (all dilutions 1:1000). Membranes were then washed with TBST 5 min and incubated with horseradish peroxide (HPR)-conjugated secondary anti-mouse or anti-rabbit antibodies (1:3000) in the presence of 5% skim milk in TBST for 1 h at room temperature. For chemiluminescence detection, the membranes were visualized using enhanced chemiluminescence ECL reagent (Amersham Pharmacia, Piscataway, NJ, USA). Band densities were calculated using image making software (ImageJ; National Institute of Health, Bethesda, MD, USA).

### 2.11. Statistical Analysis

Results are presented as means ± standard deviations (SD) from three independent replicates. The significances of intergroup differences were determined by one-way analysis of variance (ANOVA) followed by Duncan’s multiple range test using PASW statistics Ver. 18 (SPSS Inc., Chicago, IL, USA). Statistical significance was accepted for *p* values < 0.05.

## 3. Results

### 3.1. Effect of MCR on Weight Gain in HFD-Induced Mice

**Figure 1:**
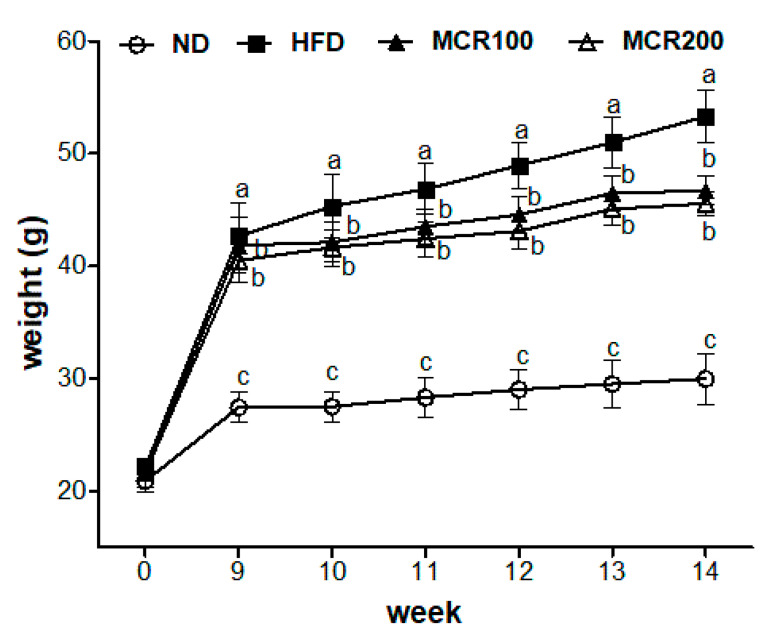
Effect of *Mori Cortex radicis* extract (MCR) on body weights of HFD-induced (High Fat Diet-Induced) mice. Mice were fed ND or HFD with or without MCR (100 or 200 mg/kg b.w.) for 14 weeks. Weights were monitored weekly. Results are presented as means ± SDs (*n* = 8). Statistical significance was accepted for *p* values < 0.05. Small letters indicate significantly different from other groups.

In order to evaluate the inhibitory effect MCR on HFD induced-weight gain, mice were fed the HFD for 8 weeks to induce obesity and then with the HFD plus MCR (100 or 200 mg/kg/day) for 6 weeks. Changes in body weights in the ND, HFD, MCR100, and MCR200 groups are shown in Figure 1. After 8 weeks on the HFD, mean weight increased by 52.0% (42.6 ± 2.8 g) versus the ND group (27.5 ± 1.3 g). After the following 6 weeks of MCR administration, mean weight in the HFD, MCR100, and MCR200 groups increased to 53.3, 46.6, and 46.4 g, respectively, which represented increases of 77.6, 55.3, and 54.7% over that of the ND group (30.0 ± 2.2 g). Furthermore, weights were significantly lower in the MCR100 and MCR200 groups than in the HFD group.

### 3.2. Effect of MCR on Glucose Tolerance in HFD-Induced Mice

**Figure 2:**
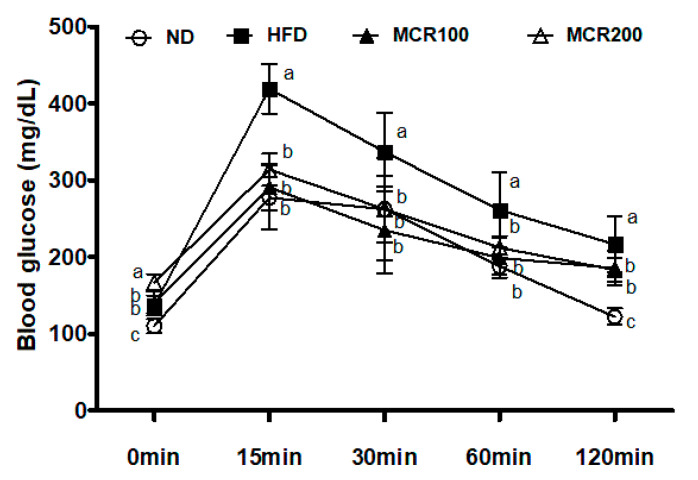
Effect of *Mori Cortex radicis* extract (MCR) on oral glucose tolerance (OGTT) in HFD-induced mice. Mice were fed ND or HFD with or without MCR (100 or 200 mg/kg b.w.) for 14 weeks. After 14 weeks of diet, mice were then fasted for 12 h, and blood glucose levels were measured during 120 min. Results are presented as means ± SDs (*n* = 8). Statistical significance was accepted for *p* values < 0.05. Small letters indicate significantly different from other groups.

In order to evaluate the effect of MCR on HFD-induced glucose intolerance, OGTT was performed after the 14-week experimental period. Longitudinal changes in blood glucose levels are shown in Figure 2. After administering glucose, blood glucose levels in the HFD group rapidly increased to 419.0 ± 32.2 mg/dL at 15 min, which was significantly greater than that observed in the ND, MCR100, and MCR200 groups (277.3 ± 40.3, 290.8 ± 29.1, and 313.6 ± 20.1 mg/dL, respectively). Continuously, the blood glucose level of the HFD group was higher than that in the three other groups at 30 and 60 min. At 120 min, blood glucose levels in the HFD group remained high at 216.5 ± 36.5 mg/dL. In contrast to the HFD group, ND, MCR100, and MCR200 groups’ blood glucose levels were lower at 122.1 ± 10.2, 185.4 ± 22.5, and 183.0 ± 15.3 mg/dL, respectively (*p* < 0.05).

### 3.3. Effect of MCR on Spatial Learning and Memory in HFD-Induced Mice

**Figure 3:**
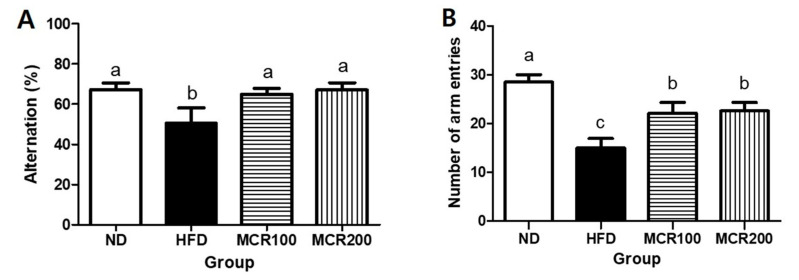
Effect of *Mori Cortex radicis* extract (MCR) on spontaneous alternation behavior in HFD-induced mice. (**A**) Spontaneous alternation and (**B**) number of arm entries. Mice were fed ND or HFD with or without MCR (100 or 200 mg/kg b.w.) for 14 weeks. At 14 weeks, Y-maze behavioral test was then performed. Results are presented as means ± SDs (*n* = 8). Statistical significance was accepted for *p* values < 0.05. Small letters indicate significantly different from other groups.

The Y-maze test is a behavioral test based on the tendency of mice to explore novel rather than familiar objects [9]. The alternation behavior of the HFD group (50.4%) was significantly less than those of ND, MCR100, and MCR 200 groups (67.2%, 65.0%, and 67.1%, respectively) (Figure 3A,B). The HFD group consecutively entered different arms 15.0 times, which was significantly lower than ND group (28.5 times), and MCR100 and MCR 200 groups entered arms more frequently than the HFD group (22.1 and 22.5 times, respectively) (*p* < 0.05).

### 3.4. Effect of MCR on Serum Parameters in HFD-Induced Mice

Lipid serum parameters are summarized in Table 1. Triglycerides (TG) contents were higher in HFD than in ND group (51.7 ± 16.8 versus 30.0 ± 7.8 mg/dL), but MCR 200 group was significantly lower than HFD group, (36.0 ± 10.9 mg/dL, no difference was found with ND group). Total cholesterol (TCHO) contents were higher in HFD, MCR100, and MCR 200 groups than in ND group (183.5 ± 27.4, 203.4 ± 16.9, and 199.8 ± 24.9 mg/dL versus 90.9 ± 5.7 mg/dL), but no significant difference was found between HFD, MCR100, and MCR 200 groups. Low density lipoprotein (LDL) contents in HFD, MCR100, and MCR 200 groups (15.7 ± 6.7, 13.2 ± 2.5, and 13.6 ± 1.7 mg/dL, respectively) were markedly higher than in ND group (3.5 ± 0.6 mg/dL), but differences between the HFD and MCR100 and MCR 200 groups were not significant. HTR (%) (defined as a percentage of high-density lipoprotein (HDL) to TCHO) was lower in the HFD group (34.4 ± 3.9%) than ND group (58.8 ± 1.7%). In comparison with the HFD, however, MCR100 and MCR 200 groups significantly increased HTR (36.9 ± 2.0 and 37.4 ± 1.4%, respectively).

### 3.5. Effect of MCR on the Antioxidative Systems of HFD-Induced Mice

**Figure 4:**
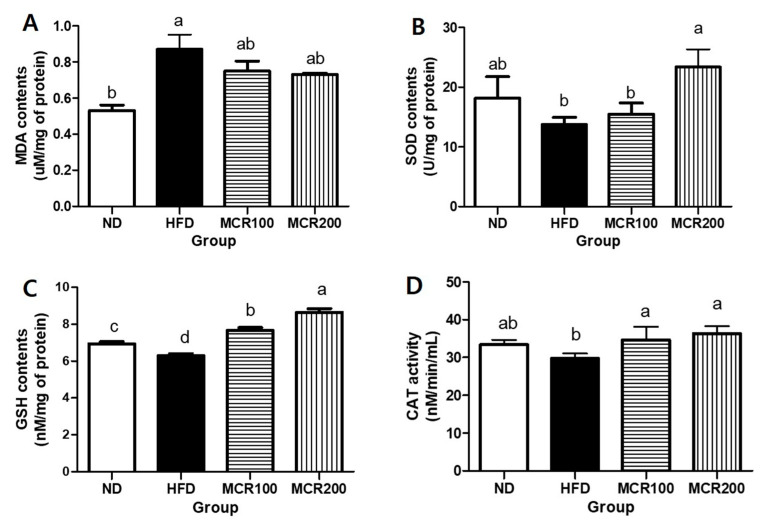
Antioxidant effect of *Mori Cortex radicis* extract (MCR) on HFD-induced cognitive dysfunction mice. Mice were fed ND or HFD with or without MCR (100 or 200 mg/kg b.w.) for 14 weeks. (**A**) malondialdehyde (MDA), (**B**) superoxide dismutase (SOD), (**C**) glutathione (GSH) contents, and (**D**) catalase (CAT) activity in mouse brain. Results are presented as means ± SDs (*n* = 8). Statistical significance was accepted for *p* values < 0.05. Small letters indicate significantly different from other groups.

Brain tissue is vulnerable to oxidative stress as it is rich in unsaturated fatty acids. When damaged by ROS, unsaturated fatty acids are oxidized, and malondialdehyde (MDA) is produced [6]. At the end of the 14-week experimental period, MDA content in the HFD was 0.86 ± 0.1 nM/mg of protein, which was higher than ND group (0.53 ± 0.5 nM/mg of protein). MDA contents of MCR100 and MCR200 groups (0.75 ± 0.1 and 0.73 ± 0.0 nM/mg of protein) were reduced (Figure 4A).

In order to investigate the anti-oxidative effect of MCR on HFD-induced mouse brain tissues, we evaluated SOD and GSH contents and CAT activity. The results are summarized in Figure 4B–D, respectively. SOD content in the HFD was lower than ND group (13.7 ± 1.2 vs. 18.1 ± 3.2 U/mg of protein). However, it was shown that SOD content of MCR200 group was significantly higher than in the HFD group (23.3 ± 2.9 U/mg of protein). GSH content in the HFD was lower than in the ND group (6.2 ± 0.2 vs. 6.9 ± 0.2 nM/mg of protein). However, MCR100 and MCR 200 groups (7.6 ± 0.2, 8.6 ± 0.3 nM/mg of protein, respectively) were significantly higher than in the HFD group. CAT activity in the HFD group was lower than in the ND group (29.7 ± 1.2 vs. 33.4 ± 1.2 nM/min/mL). However, CAT activities in the MCR100 and MCR200 groups (34.6 ± 3.4 and 36.3 ± 1.9 nM/min/mL, respectively) were significantly higher than in the HFD group (*p* < 0.05).

### 3.6. Effect of MCR on AChE Activity and Protein Expression in HFD-Induced Mice

**Figure 5:**
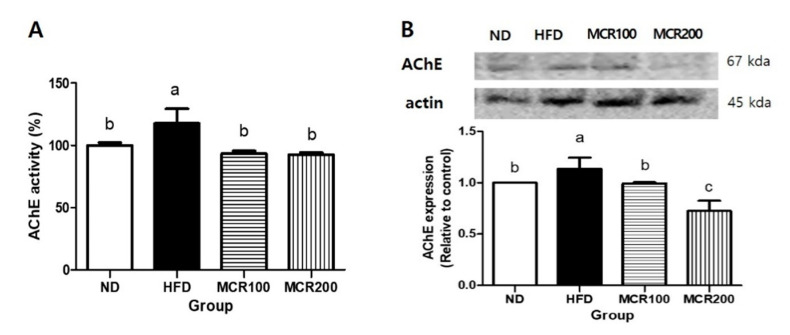
The effect of *Mori Cortex radicis* extract (MCR) on HFD-induced acetylcholinesterase (AChE) activity. (**A**) AChE activity and (**B**) its protein expression. Mice were fed ND or HFD with or without MCR (100 or 200 mg/kg b.w.) for 14 weeks. AChE activity and protein in brain tissues were measured using an ELISA kit by western blotting, respectively. Results are presented as means ± SDs (*n* = 8). Statistical significance was accepted for *p* values < 0.05. Small letters indicate significantly different from other groups.

Acetylcholinesterase (AChE) is an enzyme that hydrolyzes acetylcholine. When AChE is activated, cholinergic reactions are inhibited, which leads to memory defects [17]. To evaluate AChE regulation by MCR in the brain tissues of HFD-induced mice, we measured AChE activity and protein expression (Figure 5). AChE activity was 17.7% higher in HFD group than in ND group, but both MCR100 and MCR200 groups significantly decreased by 20.7% and 21.2% compared to HFD group. Similarly, AChE protein expression was decreased by 12.6% and 36.2% in MCR100 and MCR200 groups, respectively compared to HFD group (*p* < 0.05).

### 3.7. Effect of MCR on Insulin Receptor Signaling in HFD-Induced Mice

**Figure 6:**
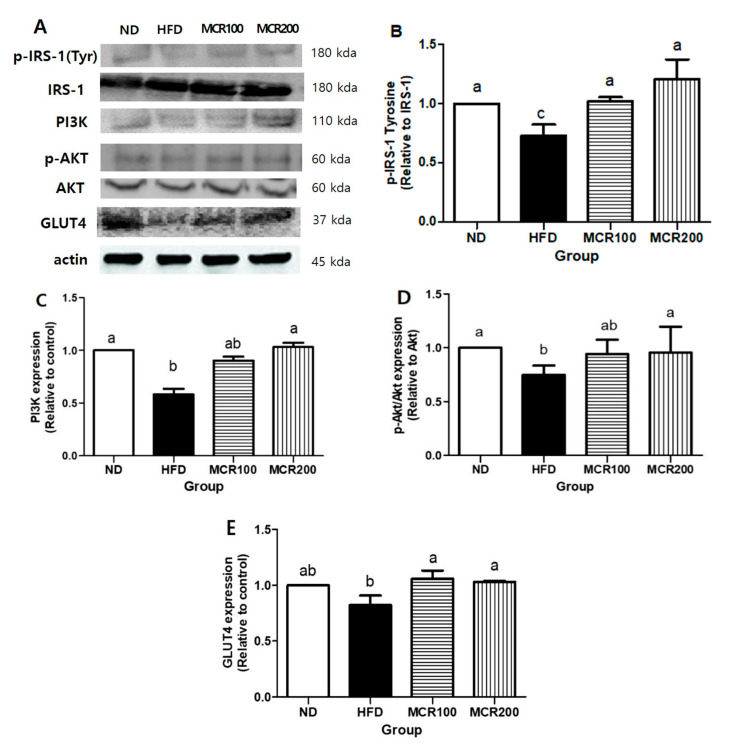
The effect of *Mori Cortex radicis* extract (MCR) on insulin receptor signaling in HFD-induced mice. Mice were fed ND or HFD with or without MCR (100 or 200 mg/kg b.w.) for 14 weeks. Protein levels in mouse brain tissues were determined by western blotting. (**A**) Representative western blots of p-IRS, PI3K, p-Akt, Akt, and GLUT4 in HFD mouse brains. Mice were fed ND or HFD with or without MCR (100 or 200 mg/kg. b.w.) for 14 weeks. Cerebral expression levels of (**B**) p-IRS, (**C**) PI3K, (**D**) p-Akt, and (**E**) GLUT4. Results are presented as means ± SDs (*n* = 8). Statistical significance was accepted for *p* values < 0.05. Small letters indicate significantly different from other groups.

The brain consumes two-thirds of body glucose, and thus, the maintenance of optimum glucose levels is important for cognition [5]. In order to investigate the effect of MCR on downstream in the insulin signaling pathway in HFD-induced mice, we evaluated the basal proteins expression levels related to the insulin receptor signaling pathway. p-IRS-1 expression was 27.4% lower in HFD group than in ND group, but its expressions were 39.9% and 60.0% higher, respectively, in MCR100 and MCR200 groups than in HFD group (*p* < 0.05) (Figure 6B). The protein expressions of PI3K, p-AKT, and GLUT4, which are downstream proteins in the insulin signaling pathway, were also evaluated. PI3K expression was 18.1% lower in HFD group than in ND group (100.0%), but 38.7% and 58.8% higher, respectively, in MCR100 and MCR 200 groups than in HFD group. p-AKT expression was 25.3% lower in HFD group than in ND group, but in MCR100 and MCR200 groups were 25.8% and 27.8% higher than in HFD group (Figure 6D). GLUT4 expression was 17.8% lower in HFD group than in ND group, but in MCR100 and MCR200 groups were 29.2% and 28.7% higher than in HFD group (*p* < 0.05) (Figure 6E).

### 3.8. Effect of MCR on Neuronal Damage in HFD-Induced Mice

It has been established that disruption of insulin signaling is a major etiology of neuronal diseases such as Alzheimer’s and Parkinson’s diseases and that brain neuronal diseases, diabetes mellitus, and obesity are closely connected, and neurodegeneration is triggered by insulin signaling [5,18].

**Figure 7:**
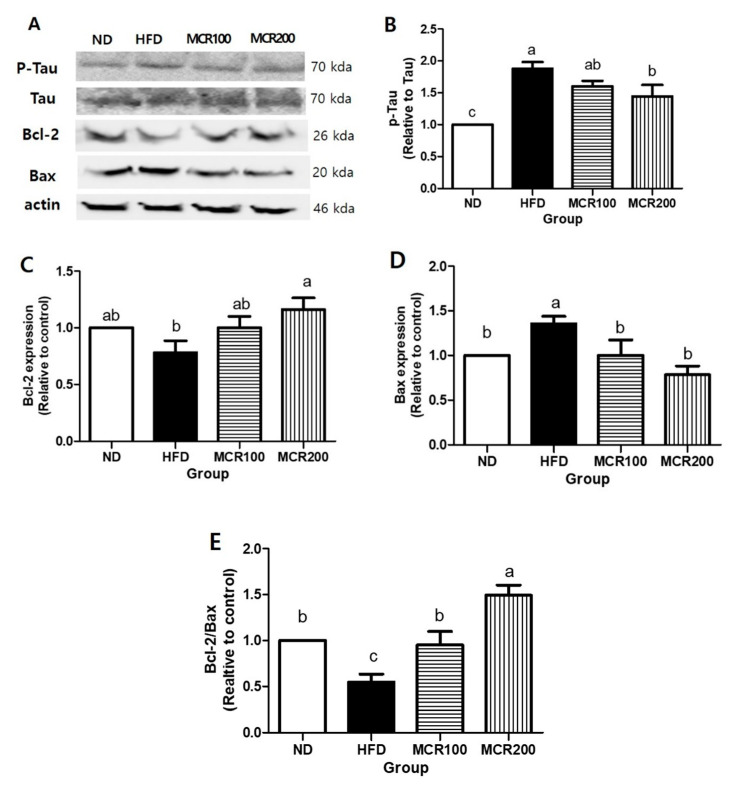
Protective effect of *Mori Cortex radicis* extract (MCR) on neuronal damage in HFD-induced mice. Mice were fed ND or HFD with or without MCR (100 or 200 mg/kg b.w.) for 14 weeks. Protein expression levels in brain tissues were measured by western blotting. (**A**) Representative western blots of p-Tau, Tau, Bcl-2, and Bax. Cerebral protein expression levels of (**B**) p-Tau, (**C**) Bcl-2, and (**D**) Bax. (**E**) Bcl-2/Bax ratios. Results are presented as means ± SDs (*n =* 8). Statistical significance was accepted for *p* values < 0.05. Small letters indicate significantly different from other groups.

In the present study, we investigated the effects of MCR on p-Tau expression and Bax and Bcl-2 (pro- and anti-apoptotic proteins, respectively). p-Tau expression was 85.2% higher in HFD group than in ND group, but 14.72 and 22.6% lower, respectively, in MCR100 and MCR200 groups than in HFD group (*p* < 0.05) (Figure 7B).

Bcl-2 expression in HFD group was 35.6% lower than in ND group, but both MCR100 and MCR200 groups showed 21.9% and 37.8% higher compared to HFD group (*p* < 0.05). Bax expression was 35.6% higher in HFD group than in ND group but MCR100 and MCR200 groups were 35.3% and 57.0% lower than in HFD group, respectively (*p* < 0.05) (Figure 7C,D). Consequently, Bcl-2/Bax ratio was 45.5% lower in HFD group than in ND group but 74.4% and 173.3% higher in MCR100 and MCR200 groups than in HFD group (*p* < 0.05) (Figure 7E).

## 4. Discussion

It is well known that long-term consumption of a high-fat diet can cause hypertension, obesity, and type 2 diabetes and also induces metabolic disorders that cause oxidative stress and damage brain tissue [3]. Metabolic diseases also exhibit insulin dysfunction, and insulin resistance in brain tissues might lead to neurodegenerative brain diseases like Alzheimer’s disease [4,5].

MCR has been reported to have anti-diabetic, hypoglycemic, and anti-depressant effects [10,19]. Previously, we reported the antioxidant and neuroprotective effects of MCR on PC12 neuronal cells cultured in glucose rich media [11]. In the present study, we evaluated the effects of MCR on memory, oxidative stress, and neuronal damage in HFD-induced group.

In this study, we used whole brain to investigate enzymes and protein expression in each group. According to the study of Liang et al. [20], insulin signaling- and stress-related proteins were expressed with similar patterns in both hippocampus and prefrontal cortex under high-fat diet conditions. In addition, Chawla et al. [21] reported that diet in high fat could impair neuronal factors in hippocampus and prefrontal cortex because both regions are responsible for learning and memory by interacting with each other.

We observed that MCR administered at 100 or 200 mg/kg significantly inhibited HFD-induced weight gain and HFD-induced disruptions of lipid metabolic markers (TG, LDL, and HDL levels). Overconsumption of dietary fat increases adipose mass and promotes adipose dysfunction, and these changes elevate free fatty acids and cause liver damage, which could impair metabolic systems and elevate LDL levels [1]. Our results suggest that MCR improves dyslipidemia caused by HFD consumption and inhibits weight gain.

It has been reported obesity is closely related to type 2 diabetes mellitus (T2DM). High calorie diets increase carbohydrate accumulation and induce high glucose conditions, and the insulin resistance detected at the onset of T2DM indicates tissues have problems utilizing insulin [22]. Insulin maintains glucose homeostasis by reducing post-prandial blood glucose concentrations, and in a background of obesity and type 2 diabetes, insulin fails to control blood glucose levels [6,7]. In the present study, OGTT testing showed blood glucose levels were markedly higher in the HFD group than in the ND group, indicating glucose intolerance. On the other hand, animals in the MCR100 and MCR200 groups exhibited lower blood glucose spikes and lower blood glucose levels than those in the HFD group. Similarly, Park et al. [23] found that *Mori Cortex radicis* extract administered for 4 weeks effectively suppressed blood glucose in an STZ-induced mouse model of diabetes.

Epidemiological studies have shown T2DM is associated with peripheral insulin resistance and central insulin signaling defects. Peripheral insulin resistance could induce insulin signaling disorders in the central nervous system and cause neuronal death and reduce synapse plasticity in the hippocampus [24]. Furthermore, these cerebral neurotransmitter dysfunctions adversely influence learning and memory [5,25]. As Figure 3 shows, MCR improved cognition and memory of HFD fed mice by increasing alternating behavior. In a study by Lee et al. [19], MCR administration ameliorated abnormal behavior and immobility and recovered neurotransmitter disfunction in brain disease induced rats.

Cognition and memory are related to the cholinergic neuronal system. Acetylcholine is produced by combining Acetyl-CoA and choline and plays critical roles in the regulation of cognition and behavior. Cohen et al. [26] demonstrated that AChE (an enzyme responsible for breaking down acetylcholine) levels are increased in insulin signaling disorders, and it has also been reported that increased AChE activity is related to acetylcholine activity and the induction of cholinergic cognitive disorder [26]. As shown in Figure 5, the administration of MCR effectively suppressed the expression of AChE, which suggests MCR might enhance cognitive and memory functions by suppressing HFD-induced cholinergic dysfunction.

Cell membranes are largely composed of phospholipids, and the brain contains relatively large amounts of lipids, which are vulnerable to oxidative stress. When brain cells are attacked by free radicals, unsaturated fatty acid, phospholipids, and lipoproteins are oxidized to lipid peroxides and malondialdehyde (MDA) [27,28], and it has been reported obesity elevates oxidative stress and impairs the antioxidative system. Furthermore, disruption of this system in neuronal tissues increases ROS levels and vulnerability to ROS. Dietary antioxidants augment antioxidative systems [28,29], and in an animal study, the administration of *Dendropanax,* which is rich in phenolic compounds, to HFD group enhanced antioxidant enzyme levels and inhibited MDA production. Kim et al. [9] demonstrated that long-term HFD consumption can trigger oxidative stress in brain cortex and hippocampus that disrupts the antioxidative system and causes MDA accumulation, neuron apoptosis and consequent cognition and memory damage. In the present study, MDA levels were up-regulated and antioxidant enzymes (e.g., SOD, GSH, and CAT) levels were down-regulated in the brain tissues of HFD group, and MCR administration reversed these changes. It has been previously reported MCR contains the prenylflavonoids, such as cyclomulberrin, morusin, sanggenonI, and kuwanonU [30] and that the prenyl group attached to the flavonoid backbone increases bioactivity by interacting with biological membranes and target proteins [31]. Much evidence supports the ROS scavenging effects of prenylflavonoids, and many plants containing prenylflavonoids are of medicinal interest (e.g., Herba epimedii, hop, and licorice) and exhibit antioxidant effects [32,33,34]. In addition, our previous study demonstrated that MCR inhibited oxidative stress and the apoptosis of neuronal PC12 cells [10,11]. Thus, we suggest the antioxidative activity of MCR may be due to the various prenylflavonoids it contains.

Chronic excessive fat and calorie consumption also increase intracellular free fatty acid levels, change cellular signaling, and induce systematic lipid disorders leading to neuronal dysfunction [1]. Obesity, which is defined as low grade inflammation in visceral adipose tissue, is a risk factor of metabolic syndrome and type 2 diabetes. Systemic metabolic disorders disrupt insulin functions and inhibit activation of insulin receptors in hippocampus [1,6]. Insulin receptors take part in phosphorylation of insulin receptor substrates, such as IRS-1 and IRS-2. These are expressed and mediate insulin action in most tissues. [35]. In fact, the amount of insulin in hippocampus, hypothalamus, and cerebral cortex is 10–100 times that in plasma. Insulin also promotes neuronal cell growth and increases synapse plasticity [36]. Thus, insulin function in the central nervous system is related to homeostasis and proper brain function [5]. The activation of insulin receptors in hippocampus increases cognition. When insulin receptors in cell surfaces are phosphorylated, tyrosine residues of insulin receptor substrate (IRS) family proteins are also phosphorylated and continuously activate phosphoinositide 3-kinase (PI3K) and Akt [12]. Activation of Akt is followed by movement of glucose to hippocampus from plasma by glucose transporters [5]. In the present study, p-IRS, PI3K, p-Akt, and GLUT4 levels were down-regulated in HFD group, which indicates deficient insulin signaling, and MCR treatment reversed this effect of the HFD.

PI3K/Akt also regulate Tau (microtubule binding protein), a key player in the pathogenesis of Alzheimer’s disease [2]. Reduced PI3K and p-Akt activate GSK3β inducing Tau over-phosphorylation. Normally, Tau regulates microtubule dynamics for cell viability and normalize axonal transport, but when Tau is over-phosphorylated, it becomes hydrophilic, and as a result, microtubules disassemble and form neurofibrillary tangles (NFTS), which cause synapse impairment and neurodegeneration [5,18,30]. In the present study, p-Tau levels were elevated in HFD group, which concurs with a report by Bhat et al. [37] that a high fat and cholesterol diet increased p-Tau expression in mouse hippocampus. On the other hand, in the present study, MCR supplementation reduced p-tau expression.

High fat consumption inhibits the functions of insulin and insulin receptors and causes insulin signaling deficits, which result in the overproduction of ROS and apoptosis due to mitochondrial dysfunction [9]. The PI3K/Akt pathway is downstream of the insulin transport pathway and simultaneously regulates cell survival and death. Inhibition of Akt increases pro-apoptotic protein expression but suppresses anti-apoptotic protein expression. Bax is a pro-apoptotic protein and dimerizes with Bcl-2 an anti-apoptotic protein [11]. In the present study, Bax levels were up-regulated and Bcl-2 levels down-regulated in HFD group, and MCR treatment effectively normalized these HFD-induced changes and, consequently, increased Bcl-2/Bax ratios. In a previous study, we examined the anti-apoptotic effect of MCR in high-glucose-treated PC12 neuronal cells [11], which agrees with the findings of the present in vivo study.

## 5. Conclusions

We investigated whether MCR protects brain function through insulin signaling in an HFD mouse model of obesity. MCR administration was found to normalize HFD-induced disruptions of lipid and glucose metabolism and to improve cognition and memory. In addition, MCR upregulated the cholinergic system and normalized antioxidative imbalance and insulin resistance. Furthermore, our results show the neuronal protective effects of MCR are due to the suppression of p-Tau production and elevation of Bcl-2/Bax ratio as regulated by insulin signaling. Therefore, we suggest MCR could be used to suppress obesity-induced metabolic cognitive diseases.

## Figures and Tables

**Table 1:** Effect of *Mori Cortex radicis* extract (MCR) on serum biomarkers in HFD-induced (High Fat Diet-Induced) mice.

Biomarker	Group			
	ND	HFD	MCR 100	MCR 200
TG (mg/dL)	30.0 ± 7.8 ^b^	51.7 ± 16.8 ^a^	39.7 ± 10.2 ^a,b^	36.0 ± 10.9 ^b^
TCHO (mg/dL)	90.9 ± 5.7 ^b^	183.5 ± 27.4 ^a^	203.4 ± 16.9 ^a^	199.8 ± 24.9 ^a^
LDL (mg/dL)	3.5 ± 0.6 ^b^	15.7 ± 6.7 ^a^	13.3 ± 2.5 ^a^	13.6 ± 1.7 ^a^
HTR (%)	58.8 ± 1.7 ^a^	34.4 ± 3.9 ^c^	36.9 ± 2.0 ^b,c^	37.4 ± 1.4 ^b^

Mice were fed ND or HFD with or without MCR (100 or 200 mg/kg b.w.) for 14 weeks, and serum samples were then collected. Results are presented as means ± SDs (*n* = 8). Statistical significance was accepted for *p* values < 0.05. Small letters indicate significantly different from other groups. Triglyceride (TG), total cholesterol (TCHO), low density lipoprotein (LDL), and HDL/TCHO × 100 (HTR).
